# The Potential of Tetrandrine as a Protective Agent for Ischemic Stroke

**DOI:** 10.3390/molecules16098020

**Published:** 2011-09-16

**Authors:** Yun Chen, Ya-Hui Tsai, Sheng-Hong Tseng

**Affiliations:** 1 Department of Surgery, Far Eastern Memorial Hospital, Pan-Chiao, New Taipei 220, Taiwan; Email: ychen@mail.femh.org.tw (Y.C.); yahuitsai@gmail.com (Y.-H.T.); 2 Department of Chemical Engineering and Materials Science, Yuan Ze University, Chung-Li, Taoyuan 320, Taiwan; 3 Department of Surgery, National Taiwan University Hospital and National Taiwan University College of Medicine, Taipei 100, Taiwan

**Keywords:** ischemic stroke, tetrandrine, alkaloid

## Abstract

Stroke is one of the leading causes of mortality, with a high incidence of severe morbidity in survivors. The treatment to minimize tissue injury after stroke is still unsatisfactory and it is mandatory to develop effective treatment strategies for stroke. The pathophysiology of ischemic stroke is complex and involves many processes including energy failure, loss of ion homeostasis, increased intracellular calcium level, platelet aggregation, production of reactive oxygen species, disruption of blood brain barrier, and inflammation and leukocyte infiltration, *etc*. Tetrandrine, a bisbenzylisoquinoline alkaloid, has many pharmacologic effects including anti-inflammatory and cytoprotective effects. In addition, tetrandrine has been found to protect the liver, heart, small bowel and brain from ischemia/reperfusion injury. It is a calcium channel blocker, and can inhibit lipid peroxidation, reduce generation of reactive oxygen species, suppress the production of cytokines and inflammatory mediators, inhibit neutrophil recruitment and platelet aggregation, which are all devastating factors during ischemia/reperfusion injury of the brain. Because tetrandrine can counteract these important pathophysiological processes of ischemic stroke, it has the potential to be a protective agent for ischemic stroke.

## 1. Introduction to Ischemic Stroke

Stroke is one of the leading causes of mortality, with a high incidence of severe morbidity in survivors [1]. 85% of all strokes are due to vascular occlusions (ischemic stroke), while 15% are primary intracerebral hemorrhage (hemorrhagic stroke) [1]. The brain is sensitive to ischemia and short periods of ischemia can cause cellular damage or death. Generally, early restoration of blood flow is important for preventing or reducing brain injury following a stroke. However, not only the ischemic insult, but also the reperfusion processes cause tissue damage, with the latter process inducing an inflammatory response that causes additional injury to the cerebral microcirculation and adjacent brain tissue [2]. The majority of stroke patients show a slow evolution of brain injury that occurs over a period of hours-to-days following the attack [1]. This period is the therapeutic window to inhibit the progression of tissue damage after ischemia and reperfusion; however, currently, few treatment options are available to minimize tissue death following a stroke [1,3]. 

The pathophysiology of stroke is complex and involves many processes, including energy failure, loss of ion homeostasis, acidosis, increased intracellular calcium levels, excitotoxicity, reactive oxygen species (ROS)-mediated toxicity, generation of arachidonic acid products, cytokine-mediated cytotoxicity, complement activation, disruption of blood-brain barrier, activation of glial cells, and inflammation and leukocyte infiltration [1]. These are interrelated and coordinated events, which can lead to ischemic necrosis in the ischemic-core regions [1]. Within a few minutes after cerebral ischemia, the core of brain tissue exposed to the most dramatic blood flow reduction is severely injured, and then undergoes necrosis and cell death [1,4]. The region around the infarct core, known as the ischemic penumbra, has less severe ischemia; and many neurons in the ischemic penumbra may undergo apoptosis after several hours or days, and thus they are potentially recoverable for some time after the onset of stroke [1,5]. 

Since stroke is associated with high mortality and severe morbidity, many studies have been devoted to investigate the treatment strategies for stroke. However, currently, the treatment to minimize tissue injury after stroke is disappointing, thus, it is mandatory to develop effective treatment strategies for stroke.

## 2. Introduction to Tetrandrine

**Figure 1:**
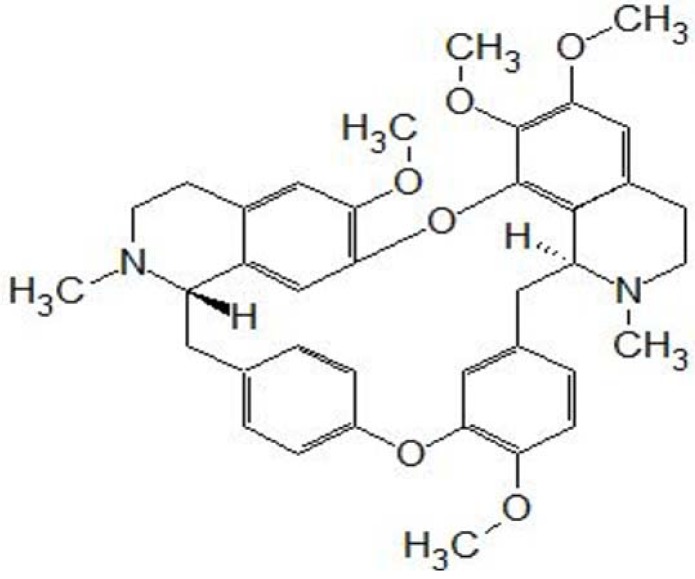
Stucture of tetrandrine.

Tetrandrine (C_38_H_42_O_6_N_2_, MW 622.730, Figure 1) is a bisbenzylisoquinoline alkaloid that is extracted from the root of *Stephania tetrandra* S. Moore, a herbaceous perennial vine of the family Menispermaceae [6]. Tetrandrine has many pharmacological effects including antiinflammatory, anticancer, immunosuppressive, and cytoprotective effects, and it has been used in traditional Chinese medicine [6,7,8,9]. It is a calcium channel blocker and has various cardiovascular effects such as reduction of portal venous pressure, antihypertensive and antiarrhythmia effects [9]. Furthermore, it suppresses the morphine-induced antinociception effects and the proliferation of vascular smooth muscle cells, and prevents diabetes mellitus [10,11,12]. Tetrandrine also exerts immunosuppressive effects, and can alleviate the graft rejection and prolong the survival of allogenic hearts in mice [13]. In addition, tetrandrine-treated dendritic cells increase the survival time of skin grafts in mice [14]. Tetrandrine exerts antitumor effects on some cancer cells, including breast, gastric, and lung cancers, neuroblastoma, Burkitt’s lymphoma, hepatoma, esophageal and colon cancers, leukemia, nasopharyngeal carcinoma, cancer cells isolated from ascites or pleural effusions, and gliomas [9]. There is also *in vivo* evidence of the anticancer effects of tetrandrine on Ehrlich ascites carcinoma and sarcoma-180 in mice, pulmonary metastatic foci in CT26 colorectal adenocarcinoma-bearing mice, human breast cancer xenografts in athymic nude mice, subcutaneous colon cancers in mice, and both subcutaneous and intracerebral gliomas in rats [8,9,15].

## 3. Effects of Tetrandrine on the Ischemia-Reperfusion Injury of Various Tissues/Organs

Tetrandrine has been found to protect various tissues/organs from ischemia/reperfusion (I/R) injury [16,17,18,19]. It could inhibit vascular contraction and improve the microcirculation of body by inhibiting the activity of phospholipase A2 in inflammatory leukocytes and the release of inflammatory mediators, and eliminating ROS [20]. Tetrandrine protects the heart from I/R injury. Myocardial I/R injury is associated with excess calcium flux into cardiomyocytes, enhanced adhesion of neutrophils to the endothelium, followed by the release of large amounts of ROS and proteolytic enzymes [17]. Tetrandrine can inhibit calcium influx into the myocyte and reduce protein release during reperfusion with a calcium containing solution following reperfusion with a calcium free solution, and arrhythmia during reperfusion in the isolated perfused heart [18]. In addition, intravenous administration of tetrandrine (0.1 and 1.0 mg/kg) 15 min prior to coronary artery ligation reduces myocardial I/R-induced lipid peroxidation of cell membrane, ROS production, neutrophil infiltration, ventricular tachyarrhythmia, myocardial infarct size, and mortality in rats [17]. Tetrandrine also exerts protective effects on liver and small bowel I/R injury [16,19]. Warm hepatic I/R induced by 50 min of ischemia followed by 24 h of reperfusion in rats increases malonyldialdehyde generation and reduces superoxide dismutase (SOD) activity in the liver tissue [16]. In such a hepatic I/R model, pretreatment with intraperitoneal injection of 50 mg/kg tetrandrine 30 min before ischemia reduces ROS and protects the liver against damage induced by I/R [16]. Furthermore, in the transplanted small bowel of the pigs, tetrandrine reduces mucosal damage, serum and tissue proinflammatory cytokine (tumor necrosis factor-α [TNF-α], interleukin-1β [IL-1β], and IL-6) levels, and intercellular adhesion molecule-1 (ICAM-1) expression and neutrophil accumulation [19]. As a whole, all the evidence suggests that tetrandrine suppresses the I/R injury in the heart, liver and small bowel. However, the effects of tetrandrine on the cerebral I/R injury or stroke are still unclear and deserve further investigation.

## 4. Calcium Channel Blocking Effects of Tetrandrine

A significant portion of ischemia-induced neuronal damage is mediated by increased intracellular calcium [21]. Calcium ions enter the cell through voltage-dependent and ligand-gated ion channels, resulting in activation of multiple signaling pathways, and a number of proteases, kinases, lipases, and endonucleases; triggering of the intrinsic apoptotic pathway and thus ending in cell death [21]. Thus, calcium channel blockers such as nimodipine and flunarazine have been used for the treatment of stroke [1]. More than 10 clinical trials of nimodipine in ischemic stroke have been conducted; however, the results are inconsistent; in addition, clinical trials with flunarizine found no statistically significant improvement in outcome of ischemic stroke [1]. Although the effects of calcium channel blockers on the ischemic stroke are unsatisfactory, investigation on other calcium channel blockers is still undergoing. Tetrandrine, also a calcium channel blocker, inhibits voltage-gated Ca^2+^ channels, large-conductance, calcium-activated potassium channels, and intracellular Ca^2+^ pumps in various types of cells such as neurohypophyseal nerve endings and vascular endothelial cells [22,23,24,25,26]. In addition to the inhibition of calcium mobilization, tetrandrine also reduces K^+^ efflux and depolarization, which are commonly noted after ischemic insult of the brain [24]. Thus, by such calcium channel blocking effect, tetrandrine might be helpful for the ischemic stroke.

## 5. Anti-Inflammatory Effects of Tetrandrine

Leukocytes play an important role in mediating reperfusion-induced tissue injury and microvascular dysfunction because they accumulate in post-ischemic tissues prior to tissue injury, neutropenia decreases the injury response to ischemic stroke, and monoclonal antibodies against leukocyte or endothelial cell adhesion molecules protect against stroke injury [27]. Therefore, polymorphonuclear leukocytes, of which neutrophils predominate, are implicated in worsening stroke outcome [27]. Reduction of circulating neutrophils can ameliorate the infarct volumes and improve the neurological outcomes in rodent [28,29]. In addition to leukocytes, T-lymphocytes and the cells within the brain including endothelial cells, astrocytes, microglia and neurons are also considered important in mediating reperfusion injury in post-ischemic brain tissue, because they secrete proinflammatory mediators and/or neuroprotective factors [2,30,31]. The protein levels of the proinflammatory cytokines, the expression of endothelial cell adhesion molecules, as well as toxic metabolites and enzymes increase in post-stroke brain tissue [32,33,34]. On the contrary, the neuroprotective factors such as erythropoietin, transforming growth factor β1, and metallothionein-2 are also increased after ischemic insult [30]. These destructive and protective factors occur at different time points following ischemic insult, and contribute to the pathophysiology of stroke in a complicated way [35,36]. 

As mentioned above, inflammation plays a significant role in the pathophysiology of stroke, thus antiinflammation treatment might be beneficial for ischemic insults. Antibodies against adhesion molecules such as anti-selectin have been found to be effective for ischemic insults in animals [37,38]; however, clinical trials using the murine anti-ICAM-1 antibody revealed worsened outcomes [39,40]. Several other anti-inflammatory cytokine approaches have also been tested in experimental stroke models, but there have been no successful clinical trials reported [1]. Tetrandrine has anti-inflammatory effect. It inhibits the functions of monocytes, macrophages, lymphocytes, neutrophils, natural killer cells and mast cells [41,42]. Furthermore, tetrandrine suppresses T and B cells and inhibits the production of cytokines and inflammatory mediators such as interleukins, prostaglandins, leukotrienes, and TNF-α, and nitric oxide [19,24,42]. *In vivo* studies found that tetrandrine could suppress the inflammatory reactions associated with rheumatoid arthritis, pancreatitis, endotoxemia, sepsis, hepatitis, bovine serum albumin-, IL-1-, or endotoxin-induced uveitis, in animals [6,20,42]. Tetrandrine inhibits the leukocyte infiltration into subcutaneous air pouches (model of inflammation) induced by IL-1 or TNF in rats, with the effective dose 50 (ED_50_, the dose of tetrandrine to suppress 50% of leukocyte infiltration) values in the range 20–30 mg/Kg/3 days [43]. Further, it significantly suppresses microvascular leakage of guinea pig airway induced by platelet-activating factor, bradykinin, leukotrienes D4 and histamine [44]. Intraperitoneal injection of 4% tetrandrine at a dose of 80 mg/kg attenuates the elevation of the expression of ICAM-1 mRNA in the pancreas and livers of rats with acute pancreatitis [20]. In addition, tetrandrine inhibits the N-formyl-methionyl-leucyl-phenylalanine or phorbol-myristate-acetate-induced rapid calcium influx, Mac-1 upregulation, accumulation of ROS, and neutrophil adhesion [45]. All the evidence indicates that tetrandrine exerts significant antiinflammatory effect, which might alleviate the devastating effects of the leukocytes and proinflammatory mediators after ischemic insults of the brain.

## 6. Antioxidative Effects of Tetrandrine

Reactive oxidative species have been considered to be closely linked to the pathophysiology of ischemic stroke [46,47,48]. The ROS involved in stroke-induced brain injury include superoxide anion radical (O_2_^.−^), hydroxyl radicals (OH) and nitric oxide (NO) [46,47,48]. The sources of ROS during ischemic stroke are the mitochondria, which produce superoxide anion radicals during the electron transport process; and the metabolism of arachidonic acid through the cyclooxygenase and lipooxygenase pathways; and NADPH oxidase, which is a major source of ROS at vascular level [46,49,50,51]. Due to their highly reactive nature, ROS react with DNA, proteins, and lipids, causing various degrees of damage and dysfunction of cellular vital structures [1]. Because of the multiple ROS-induced adverse reactions, both antioxidants and defense systems will react to keep the cells in homeostasis [52]. The cellular enzymes involved in antioxidant defense mechanisms include SOD, glutathione peroxidase, and catalase [53]. SOD and glutathione peroxidase, which are present in the cytosol and mitochondria, reduce superoxide anion to hydrogen peroxide and water, and remove the majority of hydrogen peroxide, respectively [52]. Catalase is located in peroxisomes and can remove high levels of hydrogen peroxide [52]. However, in I/R injury, these defense mechanisms may not be sufficient to counteract the effects of ROS, resulting in an imbalance of redox status [52]. Because ROS are involved in stroke-induced brain injury, thus, agents either blocking ROS production or inhibiting their activation are considered beneficial for the prevention or treatment of stroke. Although various antioxidants have been shown to be effective against experimental stroke injury [54,55]; clinically they have only limited success for the treatment of acute ischemic stroke [56,57].

Tetrandrine also has antioxidative effects [45,58]. It influences the production of ROS including hydrogen peroxide, hydroxyl radicals and superoxide radicals [23,45,58,59,60]. Tetrandrine can inhibit the lipid peroxidation. It causes a significant inhibition on freshly quartz-induced lipid peroxidation and suppresses both the initiation and the propagation of lipid peroxidation of mitochondrial membranes in rat liver mitochondrial fractions [59,61]. Tetrandrine (20 to 100 nmol/mg protein) delays the oxygen uptake burst induced by the complex ADP/Fe^2+^, and prevents ROS production, thereby decreasing the rate of lipid peroxidation of mitochondrial membranes [59]. Furthermore, pretreatment with 0.1–10 μM tetrandrine significantly decreases the hydrogen peroxide-induced elevation of glutamate release into the medium, elevation of the cytosolic free calcium concentration, generation of ROS and neuronal cell death [58]. 7.5–15 μM tetrandrine can scavenge the hydroxyl radicals generated in aqueous solution of hydrogen peroxide irradiated by ultraviolet light, and the superoxide radicals generated by the hypoxanthine/xanthine oxidase system [62]. 

In addition, tetrandrine also protects several types of cells from oxidative stress such as erythrocytes, inflammatory cells, and heart and liver tissues [45,59,63,64,65,66]. In erythrocytes, tetrandrine scavenges superoxide radicals generated from autooxidation [62]. Tetrandrine (10–1000 μg/mL) inhibits ROS generation in erythrocytes in a dose-dependent manner, and ingestion of 200 mg tetrandrine results in a significant increase in ROS scavenging in the plasma of rats [67]. Tetrandrine also reduces the production of ROS in inflammatory cells. Tetrandrine inhibits zymogen or silica-stimulated oxygen consumption, superoxide radicals release, and hydrogen peroxide secretion by alveolar macrophages in concentration-dependent manner (8–96 μM) [63,64]. It also suppresses irradiation-induced inflammatory responses, release of superoxide radicals, and phagocytic activity in normal human mononuclear cells [65]. Ultraviolet A (UVA) in rat mast cells could activate NADPH oxidase to produce ROS, which in turn activate phospholipase C signaling and trigger cytosolic calcium oscillation, while tetrandrine could abolish such effects [68]. Pretreatment with tetrandrine (1–10 μg/mL) diminishes N-formyl-methionyl-leucyl-phenylalanine- or leukotriene B4-induced intracellular ROS production (hydrogen peroxide and superoxide radicals) of neutrophils, which then suppresses the neutrophil adhesion [66]. Intraperitoneal injection of 4% tetrandrine at a dose of 80 mg/kg attenuates the reduction of the expression of SOD mRNA (Mn-SOD and Cu, Zn-SOD) in the pancreas and livers of rats with acute pancreatitis [20]. Taken together, all the data indicate that tetrandrine exerts significant antioxidative effects, which might protect the brain from the ischemic-stroke injury.

## 7. Tetrandrine Inhibits Platelet Aggregation

Chronic atherosclerotic disease is often clinically silent and coexists across vascular beds, but when complicated by thrombosis can result in ischemic stroke [69]. Platelets are key mediators of atherosclerosis and play an important role in the development of chronic atherosclerotic disease [69]. Platelet adhesion, activation and aggregation on the exposed subendothelial extracellular matrix are essential for hemostasis, but may also lead to occlusion of diseased vessels [70]. Therefore, antiplatelet agents such as acetylsalicylic acid and clopidogrel have been used for primary and secondary prevention of cerebrovascular diseases [69]. Tetrandrine can also inhibit platelet aggregation. It suppresses arachidonic acid liberation in response to collagen or thrombin, and inhibits the aggregation of rabbit platelet induced by collagen, thrombin and arachidonic acid [71]. Furthermore, tetrandrine significantly inhibits platelet aggregation induced by platelet-activating factor, with effective concentration 50 (EC_50_, the concentration of tetrandrine to suppress 50% of platelet aggregation) of 28.6 ± 3.2 μM [72,73]. However, tetrandrine does not show any anticoagulation activity in the measurement of the activated partial thromboplastin time, prothrombin time and thrombin time using human-citrated plasma [74]. *In vivo* study found that intraperitoneal administration of 50 mg/kg tetrandrine inhibited the thrombosis induced by collagen plus epinephrine in mice by 55%, while 50 mg/kg acetylsalicylic acid, a positive control, showed only 30% inhibition [74]. From these data, tetrandrine suppresses platelet aggregation and might be used as antiplatelet agent for the prevention of ischemic stroke.

## 8. Evidence of Tetrandrine against Stroke

Because tetrandrine is a highly lipid-soluble and hydrophobic molecule with a low molecular weight, it could thus cross the blood brain barrier [75]. In cultured rat cerebellar granule cells injured by hydrogen peroxide, tetrandrine (0.1–10 μM) significantly decreases glutamate release into the medium, cytosolic free Ca^2+^ concentration, generation of ROS, and cell death [58]. In the gerbil model of cerebral ischemia induced by 10-min occlusion of bilateral carotid arteries followed by 5-min reperfusion in gerbils, pretreatment with tetrandrine (15 mg/kg, intravenous infusion) enhances the recovery of electroencephalogram amplitude, reduces the calcium and water contents, attenuates the increased lipid peroxide content, and diminishes the ultrastructural abnormalities of the cortex and hippocampus in brain during I/R [76]. Although reports about the effects of tetrandrine on the stroke are limited; however, tetrandrine could protect the liver, heart, and small bowel from I/R injury, and, in addition, it counteracts some important pathophysiological processes of ischemic stroke as mentioned above, thus, it has the potential to be a protective agent for ischemic stroke.

## 9. Toxicity of Tetrandrine

The toxicity of tetrandrine depends on the dose, route and rate of administration [9]. Up to 150 mg/kg/day of tetrandrine can be used via oral or intraperitoneal routes without causing significant side effects [9]. In contrast, intravenous injection of 3–150 mg/kg tetrandrine induces significant toxic effects in beagle dogs and rhesus monkeys [77,78]. In addition, rapid intravenous injection of tetrandrine (>1 mg/kg/min) causes irregular bradycardia, cyanosis, extraventricular arrhythmias, hypotension, and even death in animals [77]. The toxicities of tetrandrine are mainly from the tetrandrine’s negative inotropic and chronotropic effects on the heart, although they may also involve the liver, kidney, and lymphoid tissues [9]. Toxic cardiovascular doses of tetrandrine decrease peripheral vascular resistance and cause vasodilatation; reduce coronary perfusion pressure, heart rate and cardiac contractility; decrease the blood pressure, induce heart block and arrhythmias, and even cause death [18,77,78]. Whenever there is prominent cardiovascular toxicity, strategies to reverse the calcium channel blockade should be used since tetrandrine is a calcium channel blocker [9].

## 10. Conclusions

In acute ischemic stroke, complex interactions among the brain endothelial cells, extravascular central nervous system cells (astrocytes, microglia, neurons), and intravascular cells (platelets, leukocytes) develop to induce ischemia/reperfusion injury through multiple processes or factors [1,27]. All these processes or factors may be adopted as the targets for developing treatment strategies. However, on the basis of the complexity of events in cerebral ischemia and the disappointing results from human clinical stroke trials using single agent, it is unrealistic to expect that a single neuroprotective agent will have benefits in human stroke [1]. Thus, a new approach targeting multiple pathogenic events of stroke may be a better strategy. 

Tetrandrine is a calcium channel blocker, and can inhibit lipid peroxidation, reduce generation of reactive oxygen species, suppress the production of cytokines and inflammatory mediators, and inhibit neutrophil recruitment and platelet aggregation, which are all devastating factors during ischemia/reperfusion injury of the brain [45,58,79,80]. Because it can counteract these important pathophysiological processes of ischemic stroke, thus, it has the potential to be a protective agent for ischemic stroke. However, more pre-clinical studies and clinical trials to assess the therapeutic effects of tetrandrine in stroke are mandatory. The time points of therapeutic manipulations are also important for the treatment of ischemic stroke. Most studies show that the therapeutic manipulations generally work best when administered before, or immediately after the ischemic insult [1]. For the treatment after insult, most effective therapies work best within 15–30 min of the stroke; rarely are they effective after more than 3 h from onset of injury [1]. Therefore, it is important to study the effects of tetrandrine administered at various time points before, during, or after ischemic insults. In addition, the balance between the safety and efficacy of tetrandrine in patients with stroke should be determined before any clinical application.
